# An Update on the Laboratory Diagnosis of *Rickettsia* spp. Infection

**DOI:** 10.3390/pathogens10101319

**Published:** 2021-10-13

**Authors:** Adam G. Stewart, Alexandra G. A. Stewart

**Affiliations:** 1 Centre for Clinical Research, Faculty of Medicine, Royal Brisbane and Women’s Hospital Campus, The University of Queensland, Brisbane, QLD 4029, Australia; 2 Department of Infectious Diseases, Royal Brisbane and Women’s Hospital, Brisbane, QLD 4029, Australia; 3 Central Microbiology, Pathology Queensland, Royal Brisbane and Women’s Hospital, Herston, QLD 4029, Australia; 4 Infectious Diseases Unit Footscray Hospital, Western Health, Melbourne, VIC 3011, Australia; alexandra.stewart@wh.org.au

**Keywords:** *Rickettsia*, spotted fever group, molecular diagnosis, metagenomics

## Abstract

*Rickettsia* species causing human illness are present globally and can cause significant disease. Diagnosis and identification of this intracellular bacteria are challenging with many available diagnostic modalities suffering from several shortcomings. Detection of antibodies directed against *Rickettsia* spp. via serological methods remains widely used with a broad range of sensitivity and specificity values reported depending on the assay. Molecular methods, including polymerase chain reaction (PCR) testing, enables species-specific identification with a fast turnaround time; however, due to resource requirements, use in some endemic settings is limited. Reports on the use of next-generation sequencing (NGS) and metagenomics to diagnose *Rickettsia* spp. infection have been increasing. Despite offering several potential advantages in the diagnosis and surveillance of disease, genomic approaches are currently only limited to reference and research laboratories. Continued development of *Rickettsia* spp. diagnostics is required to improve disease detection and epidemiological surveillance, and to better understand transmission dynamics.

## 1. Introduction

The first *Rickettsia* spp. was isolated in 1909 by Ricketts [1]; however, despite significant advances in diagnostics, infections caused by rickettsiae remain challenging to identify. Rickettsiae are obligate intracellular Gram-negative bacteria, transmitted via an arthropod vector (tick, fleas, lice or mites) from wild or domestic vertebrate hosts. The genus *Rickettsia* is classified into two major groups: the spotted fever group (SFG) and the typhus group (TG). More than 30 species are included in the SFG; such as *R. rickettsii* (Rocky Mountain Spotted Fever (RMSF)) [2], *R. conorii* (Mediterranean Spotted Fever) [3], *R. africae* (African Tick Bite Fever), and *R. australis* (Queensland Tick Typhus) [4,5]. The TG rickettsiae include *R. typhii* (murine typhus) and *R. prowazekii* (epidemic typhus) [6]. Rickettsial infections occur worldwide, with the geographic distribution of each species dependent on the vector, natural host, and climate [7]. An increasing incidence of rickettsial infections has been reported globally and the geographic distribution is expanding [5,8,9]. Due to the interplay between humans, vector, and natural host, rickettsial infections often occur in rural and remote areas. Rickettsial infections are an important cause of undifferentiated febrile illness in endemic settings but are frequently unrecognised [10,11,12]. Fever and seroprevalence studies have demonstrated a significant burden of rickettsial disease globally [13]; however, they remain a neglected disease [14]. 

Rickettsiae are introduced into the skin and spread via the lymphatic and circulatory systems to the systemic and pulmonary circulations [15]. From here, they seek to attach to their target cell. For the majority of *Rickettsia* spp., the target cell is the endothelial cell; however, *R. akari* is known to target the macrophage [16]. *Rickettsia* spp. escape the phagosome and proliferate intracellularly [17]. *R. akari* is able to disseminate via circulating macrophages, whereas other *Rickettsia* spp. achieve rapid cell-to-cell spread through hundreds of contiguous infected endothelial cells [18]. This results in a wide spectrum of disease, from a self-limiting febrile illness to life-threatening, multi-organ failure [19,20]. In addition, the intracellular location of *Rickettsia* spp. makes direct organism detection difficult in the laboratory. Clinical features include fever, headache, myalgia, and rash. An eschar may develop at the site of inoculation and provide a diagnostic clue; however, the development of an eschar varies in incidence depending on the *Rickettsia* species [11]. In severe disease, complications may include renal failure, myocarditis, meningoencephalitis, pneumonitis, acute respiratory distress syndrome, and purpura fulminans [21]. In part, disease severity depends on the causative *Rickettsia* species and their associated virulence factors-RMSF and epidemic typhus lead to a more severe disease course, whereas African tick bite fever is typically a mild disease [20]. Host factors, such as older age, co-morbidities (e.g., diabetes and alcoholism), and glucose-6-phosphate dehydrogenase deficiency, also influence disease severity [20,22]. Anti-rickettsial antibiotics are highly effective when commenced early in the disease course [23], highlighting the importance of prompt diagnosis. 

## 2. Current Challenges in Diagnosis

Both the clinical and laboratory diagnoses of rickettsial infections are challenging, which can lead to a lack of recognition or delay in diagnosis [21]. Syndromic diagnosis is problematic due to the non-specific clinical features, which may be attributed to a viral infection; bacterial sepsis; or another infectious disease endemic to the region, such as malaria, dengue, typhoid, or leptospirosis [10,22]. When a rickettsial infection is considered within the differential and anti-rickettsial antibiotics are commenced, defervescence within 48 h is often used as a diagnostic test [22]. However, a significant proportion of patients with confirmed rickettsial infections may have persisting fevers past this time point, particularly in severe disease [24]. Laboratory diagnosis relies heavily on serology, with interpretation of results dependent on appropriate epidemiology, a clinically compatible illness, and the phase of rickettsial disease when testing occurs [22]. Serological evidence of rickettsial infection does not become apparent until the second week of disease [22,25]. Hence, in the first seven days after symptom onset, when patients are most likely to present for medical care, serology is typically negative. A confirmed serological diagnosis requires acute and convalescent serology, demonstrating a fourfold rise or greater in titres. In many settings, obtaining convalescent serology at 10–14 days after symptom onset does not occur, as most patients have recovered by this time and no longer require medical care. When a single serological sample is obtained, interpretation of results is challenging and must be carefully correlated with the time from symptom onset. A non-reactive or low-titre result does not exclude a diagnosis of rickettsial infection if the sample is taken within the first seven days of illness. A reactive result, particularly of high titre in the second week of illness, may be indicative of a rickettsial infection but does not provide a definitive diagnosis. Hence, other causes of reactive rickettsial serology, such as long-term persistence of antibodies from a previous infection or cross-reactivity with another pathogen, must also be considered [22]. Molecular diagnostic tests are able to detect rickettsiae in the acute phase of illness; however, the cost and laboratory expertise required preclude their use in many endemic settings [21]. In settings with sophisticated laboratory facilities, molecular techniques may provide an accurate diagnosis when a rickettsial infection is considered within the differential early in the illness. The combined limitations of current serological and molecular diagnostics mean that diagnosis in the acute phase remains elusive, and empirical treatment is often given without any subsequent confirmation of diagnosis. The challenges in diagnosis perpetuate the neglected nature of rickettsial infections-without confirmation of cases, the true burden of disease is underestimated, leading to lack of development in diagnostics and ongoing under appreciation of clinical significance [14].

## 3. Serology 

Serology has been the mainstay for diagnosis of rickettsial infections since 1916, with the development of the Weil-Felix test [26]. Advancements in serological assays have provided greater sensitivity and specificity; however, they remain an imperfect diagnostic tool. Currently, the indirect immunofluorescence assay (IFA) is the reference-standard serological assay, however, due to cost, laboratory equipment, and technical expertise required, other assays remain in use. 

The Weil-Felix test (WFT) is a non-specific heterophile agglutination reaction, utilising cross-reactivity between rickettsiae and various *Proteus* serotypes (*P. vulgaris* OX-19 antigen (Ag)-TG antibody (Ab); *P. vulgaris* OX-2 and OX-19 Ag-SFG Ab) for detection of anti-rickettsial antibodies [25]. Agglutinating antibodies, mainly IgM, are detectable 5–10 days after symptom onset [25]. Poor sensitivity and specificity of the WFT have been demonstrated for all rickettsial groups (Table 1), and the test is now rarely used; however, in some resource-limited settings, this remains the only test available [13,21,25,27]. As serological techniques developed, complement fixation and latex agglutination tests were used, but these have now largely been replaced by IFA [28].

The indirect immunofluorescence assay is performed using fluorescein-labelled conjugate to detect serum antibodies to rickettsial antigens fixed to a slide [22]. The majority of laboratories test perform IgG IFA, as IgM antibodies do not appear significantly earlier and are less specific [22]. High rates of false positivity have been demonstrated with anti-*R. conorii* IgM, due to reaction with non-specific lipopolysaccharides [29] and similar immunogenic false-positive IgM results occur with *R. rickettsii* and other SFG rickettsiae [30]. However, IFA IgG assays demonstrate high sensitivity (83–100%) and specificity (91–100%) from the second week of illness onwards, for both SFG and TG infections (Table 1) [28,31,32,33,34]. Results are dependent on the antigens used in the assay; commercially, a limited number of established species (e.g., *R. rickettsii* or *R. conorii*) are often included, and cross-reactivity between SFG rickettsiae is utilised to facilitate group-level diagnosis [22,35]. Identification to the species level for SFG infections is typically only available in rickettsial reference laboratories, where in-house IFA, microimmunofluorescence, Western blot, and cross-adsorption assays can be performed [7,35]. Cross-reactivity also occurs between SFG and TG rickettsiae [7,22], which may preclude group-level identification. Results from IFA are reported quantitatively as titres; however, reading of slides is prone to subjectivity. Additional limitations include the need for a fluorescence microscope, with corresponding laboratory facilities and expertise. An alternative to IFA, utilising a similar technique, is the indirect immunoperoxidase test (IIP). The use of peroxidase instead of fluorescein facilitates the reading of slides by light microscopy [25], and, consequently, IIP can be performed in less sophisticated laboratories.

Microimmunofluorescence (MIF) allows for simultaneous detection of multiple rickettsial antigens in a single well [7,25]. This can assist in differentiation of species; if a species demonstrates a fourfold higher dilution compared to others, this may be suggestive of a causative organism [35]. However, this is not definitive, as, once again, cross-reactions can hinder this technique. Western blotting and cross-adsorption assays may overcome this issue. Western blot analysis allows detection of both non-specific lipopolysaccharide (LPS) and species-specific surface protein antigens (SPA), facilitating species-level diagnosis [25,36]. Cross-adsorption assays further increase specificity by demonstrating removal of homologous and heterologous antibodies when the patient serum is incubated with antigens of the causative species [25,36]. However, these methods are expensive, require technical expertise, and are limited to reference laboratories [25].

Enzyme-linked immunosorbent assay (ELISA) for IgM or IgG for SFG and TG rickettsial infections is widely used and is better suited to low-resource settings due to the ability for batch testing and reduced need for technical expertise [21,22,35]. High sensitivity and specificity have been demonstrated in all rickettsial groups (Table 1) [21,35]. Optical density (OD) readers eliminate reader bias but have the disadvantage of only providing qualitative results (reactive or non-reactive). Typically, an OD ≥ 0.5 is used as a diagnostic cut-off; however, independent validation is lacking [35]. Recently, quantitative ELISA has been developed for SFG rickettsial infections, moving away from arbitrary OD cut-offs and instead calculating cut-offs directly from negative controls, with final results reported in titres [37]. Together, these advancements allow for more accurate and objective interpretation of results [37].

Further issues with serological diagnosis include the lack of standardisation for IFA cut-off values or the antibody isotypes used (IgM, IgG, or whole Ab) [38,39,40,41]. For example, in the diagnosis of murine typhus, the IgG positivity cut-off titre ranges from ≥ 1:40 in Spain to > 1:960 in Nepal [38]. Determining accuracy for positive and negative results is dependent on an understanding of background immunity in endemic and non-endemic settings [21]. Anti-rickettsial antibodies can remain detectable for months (IgM) to years (IgG) after an infection, which makes it challenging to differentiate acute infection from a sub-clinical infection or previous exposure [22]. In endemic settings, higher diagnostic cut-off values have been suggested, with locally validated positivity cut-off values where possible [38,41]. Region-specific ELISA OD diagnostic cut-offs based on local epidemiology are also required. The clinical importance of establishing appropriate diagnostics cut-offs cannot be understated. If the cut-off is too low for the epidemiological context, there will be high numbers of false-positive results, with overdiagnosis and unnecessary treatment ensuing. However, if the cut-off is too high, then patients with rickettsial infections may remain undiagnosed and at risk of severe disease and life-threatening complications.

Improvement in current serological tests requires attention, in conjunction with further development of accurate point-of-care tests, to enable use of diagnostics where they are most needed in rural and remote settings. Progress has been made with rapid diagnostic tests for scrub typhus; however, further development is required for SFG and TG rickettsial infections [35].

**Table 1:** The comparison of sensitivity and specificity of serological tests for diagnosis of spotted fever group rickettsioses and murine typhus.

Disease	Serological Assay	Sensitivity (%)	Specificity (%)	References
Spotted fever group rickettsioses	WFT	21–70	46–96	[31,41,42,43,44]
	IFA IgM	83–85	100	[31,33]
	IFA IgG	85–100	99–100	[28,31,32,33]
	ELISA IgM	91–100	94–100	[32,42]
	ELISA IgG	83–100	87–100	[32,41]
Murine typhus	WFT	56–81	96–98	[45]
	IFA IgM	53–100	91–99	[28,34]
	IFA IgG	83–100	91–100	[28,34]
	ELISA IgM	45–98	91–98	[34]
	ELISA IgG	100	83	[34]

WFT: Weil-Felix test; IFA: immunofluorescence assay; ELISA: enzyme-linked immunosorbent assay.

## 4. Direct Detection of *Rickettsia* Species from Clinical Specimens

Nucleic acid amplification tests (NAAT) and immunohistochemistry (IHC) assays are available to aid in the direct detection of *Rickettsia* spp. (Table 2) [7,46]. This has enabled identification to species level, although for clinical purposes, species-specific diagnosis may not be necessary. IHC has the advantage of being able to be performed on formalin-fixed tissue specimens; it has been used to detect *R. rickettsii*, *R. conorii*, *R. africae*, *R. typhi*, *R. prowazekii,* and *R. akari* [47,48,49,50]. Typically, monoclonal antibodies targeting the lipopolysaccharide have been used to detect rickettsiae in IHC testing [51]. Eschar tissue is a useful specimen used for detection by IHC of rickettsiae commonly associated with this skin lesion (*R. akari, R. parkeri, R. conorii*, and *R. africae*) [50,52]. Care should be taken when handling and processing clinical specimens for IHC, as denaturation of species-specific antigens has occurred, which may lead to false-negative results. Detection of *R. conorii* in circulating endothelial cells has been achieved [53]. This method uses magnetic beads, which are coated with monoclonal antibodies directed against human endothelial cell surface antigens; an immunofluorescent stain is then performed to detect intracellular rickettsiae.

NAAT has been used to identify numerous *Rickettsia* species directly from clinical specimens [7,54,55]. Patient specimens have included blood, buffy coats, plasma, tissue (fresh, frozen, and paraffin-embedded), and swabs from the base of ulcers [56,57,58]. Many different molecular probes and primers have been used to identify *Rickettsia* DNA, including 16S rRNA gene, *gltA* (citrate synthase), *ompA* (outer membrane protein A), *sca0, sca4* and *sca5* (outer membrane proteins), HSP60, and genes encoding lipoproteins (17-kDa antigen) [7,21,59]. *Rickettsia* organisms can be detected during the early phase of illness, including in patients who are treated with antibiotics, with NAAT being slightly more sensitive when compared to isolation and cultivation. Conventional, nested, and real-time PCR techniques have all been utilised for detection [60]. *Rickettsia* DNA present in blood is likely limited in quantity and short-lived in acute infection, making clinical sensitivity of the assay suboptimal. This is mainly due to most *Rickettsia* spp. infecting endothelial cells and not circulating blood cells. In animal models, detection of rickettsial DNA in the blood occurs only intermittently, is short in duration, and is greatly affected by initiation of antibiotic therapy [61]. In one study evaluating a genus-wide NAAT for the detection of *Rickettsia* spp. from whole blood samples, sensitivity ranged from 6 to 69% when compared to the gold standard (serological diagnosis) [62]. A high volume of *Rickettsia* spp. DNA detected from blood may correlate with severe or fatal outcomes; this was especially true in *R. rickettsii* infections [63]. Sensitivity of *Rickettsia* PCR is far greater on tissue samples when compared to blood; this is particularly true in eschar samples where sensitivity can be as high as 92% [64]. Frequency of eschar has been noted to be < 1, 72, and 32–95% for RMSF, Boutonneuse fever, and African tick bite fever, respectively [65,66]. Grouped and nested PCR assays used on clinical samples to distinguish spotted fever group and typhus group *Rickettsia* spp. have been developed and used for many years and have demonstrated good sensitivity and specificity [67,68,69]. A limitation of this approach is the lack of speciation, which is often useful for clinical management and epidemiological purposes.

**Table 2:** The comparison of sensitivity and specificity of immunologic detection, molecular methods, and isolation of *Rickettsia* spp.

Assay Type	Specimen	Species	Sensitivity (%)	Specificity (%)	References
Immunohistochemistry	Skin	*R. rickettsii*, SFG rickettsioses	70	100	[47,48]
Immunohistochemistry	Eschar	*O. tsutsugamushi*	65	100	[50]
Immunocytochemical	Blood (circulating endothelial cells)	*R. conorii*	50	94	[53]
PCR	Blood	*R. rickettsia*	6–69	90–100	[54,55]
PCR	Eschar	*R. rickettsia, R. parkeri*	70–92	95–100	[50,52]
Isolation	Plasma, buffy coat, skin	*R. conorii, O. tsutsugamushi*	59	-	[55]

PCR: Polymerase chain reaction; SFG: spotted fever group.

## 5. Genomic Approaches in the Diagnosis of *Rickettsia* Species Infection

Direct detection of *Rickettsia* species through PCR methods relies on the identification of a single or limited number of pathogens through individual primers or probes [7,55]. By sequencing and characterising all DNA or RNA within a clinical specimen, metagenomic approaches have become useful in the laboratory diagnosis of infectious diseases [70,71]. Improved speed, accuracy, and cost of next-generation sequencing (NGS) platforms have largely been the driving factors for implementing this method in clinical laboratories. In addition to characterising microbial communities, metagenomics is able to perform well in identifying pathogens that are intracellular, difficult to culture, or may not present their molecular target (e.g., partial DNA sequences) [72]. Acute infection due to *Rickettsia* spp. is often difficult to diagnose due to a multitude of factors, including a transient bacteraemia phase of illness, requirement of cell culture methods for isolation, poor sensitivity of serology early in disease, and use of effective empirical antibiotics.

Reports of the successful use of clinical metagenomics in the diagnosis of *Rickettsia* spp. infection have been growing. Deep sequencing methods on blood have been used to diagnose *Rickettsia honei* infection in a middle-aged woman with fever, rash, and septic shock [73]. Standard clinical testing did not identify an infectious aetiology and serologic tests for *Rickettsia* were negative; despite administration of doxycycline, the patient died rapidly. Metagenomic analysis performed on an eschar biopsy was able to identify *Rickettsia sibirica* subsp. *sibirica* in a 50-year-old male from Qinghai Province, China [74]. The surgically excised eschar was rich in *Rickettsia* DNA recovered from high-throughput sequencing; 85% of rickettsial unique reads (226/266 (85%)) were 100% identical to *R. sibirica* subsp. *sibirica*. A cohort of 10 patients with murine typhus infection underwent NGS of microbial cell-free deoxyribonucleic acid (mcfDNA) to identify *R. typhi* [75]. Sequencing of *R. typhi* mcfDNA was more rapid and specific than serology and was able to change clinical management in half of the cases. NGS has also been used to detect *R. typhi* and diagnose murine typhus in two pregnant women [76]. *R. typhi* DNA was detected in both cases using a commercial assay (The Karius^®^ Test). A high-throughput 16S V1-V2 rRNA gene-based metagenomics assay used for detection of bacterial tick-borne pathogens has been developed [77]. This assay performed well in accurate identifying *Rickettsia* species. Outside of the clinical diagnosis of *Rickettsia* spp. infection, metagenomic sequencing of ticks has also been able to identify a range of tick-borne pathogens; this includes those with pathogenic potential that have yet to be associated with human disease [78,79].

The value of DNA sequence data from *Rickettsia* species causing human infection holds potential to extend beyond diagnosis. The epidemiology and distribution of pathogenic *Rickettsia* spp. among humans and animals have relied on seroprevalence surveys, which are fraught with error and give a low-resolution depiction of the burden of disease [80,81]. Amplicon-based NGS has been used for entomological surveillance of spotted-fever group *Rickettsia* spp. in Thailand [82]. Metagenomic sequencing of samples obtained from vectors, humans, and animals will be able to provide a more accurate illustration of the prevalence of individual *Rickettsia* spp., their transmission dynamics, and species most likely to cause disease in humans; discovery of novel species will also be facilitated through this approach. Genomic data can also be used to identify important antibiotic resistance genes, such as those that confer resistance to macrolide and tetracycline antibiotics [83].

## 6. Barriers and Implementation of Contemporary Testing Strategies

Despite the development of sequencing technology and targeted molecular assays, barriers remain with regards to their utility and implementation in the clinical setting. For NGS and metagenomic approaches, a key limitation is the reduced sensitivity caused by a high background (i.e., human DNA reads detected in clinical samples) [70]. Specimens typically used to help identify *Rickettsia* spp. (e.g., skin, eschar, and blood) are rich in human DNA. Depletion methods used to extract residual background human DNA have been developed; however, no standardisation exists [84]. Microbial enrichment methods using differential lysis of human cells followed by degradation of background DNA have been shown to reduce the detection of free nucleic acid from dead organisms lysed in vivo [85]; this may be particularly problematic for intracellular bacteria such as *Rickettsia* spp. In addition, use of genomic assays requires highly trained laboratory or personnel and currently lack well-characterised reference standards. Although PCR and other molecular approaches are more commonplace and require less infrastructure and expertise, they are not without flaws. They have reduced sensitivity outside of the acute phase of illness, which may be short lived, especially in mild disease. Available assays typically only target a limited number of important species, with utility highly dependent on geographical region. Some assays identify *Rickettsia* spp. Based on group (i.e., spotted-fever group versus typhus group). Moreover, nested conventional PCR methods have been found to be prone to amplicon contamination.

Given the geographical distribution of rickettsioses, existing resource allocation towards *Rickettsia* spp. diagnostics becomes a major limiting factor [8,86]. Indeed, rickettsioses cause a large burden of disease in developing countries, including those in sub-Saharan Africa, Southeast Asia, and South America [9,87]. The resources required for contemporary *Rickettsia* spp. diagnostics are unlikely to be delivered to such areas.

## 7. Conclusions

*Rickettsia* species causing human infection have a worldwide geographical distribution with an increasing incidence of disease. Diagnosis remains challenging, particularly in low-resource settings when relying solely on clinical manifestations. The use of older methodologies such as serology is problematic and may not reflect the true burden of rickettsial disease. Moreover, diagnosis often requires collection of convalescent serum, which is impractical in many settings. Targeted approaches in the detection of *Rickettsia* spp. have been available for some time (e.g., PCR and IHC) but are limited by the basic infrastructure required and the number of *Rickettsia* spp. able to be identified in a basic clinical laboratory. Genomic sequencing platforms are becoming cheap, fast, and more readily available. Clinical metagenomics has been used to successfully identify *Rickettsia* spp. from clinical specimens. Unfortunately, the resources and expertise required to perform these novel testing strategies are limited to reference and research laboratories at present.
